# Effects of elevated seawater *p*CO_2 _on gene expression patterns in the gills of the green crab, *Carcinus maenas*

**DOI:** 10.1186/1471-2164-12-488

**Published:** 2011-10-06

**Authors:** Sandra Fehsenfeld, Rainer Kiko, Yasmin Appelhans, David W Towle, Martin Zimmer, Frank Melzner

**Affiliations:** 1 Department of Biological Sciences, University of Manitoba, Winnipeg, MB, Canada R3T2N2; 2 Biological Oceanography, Leibniz-Institute of Marine Sciences (IFM-GEOMAR) Kiel, Düsternbrooker Weg 20, 24105 Kiel, Germany; 3 Marine Ecology, Leibniz-Institute of Marine Sciences (IFM-GEOMAR) Kiel, Düsternbrooker Weg 20, 24105 Kiel, Germany; 4 Center for Marine Functional Genomics, Mount Desert Island Biological Laboratory, Salisbury Cove, ME 04672 USA; 5 Paris-Lodron-Universität Salzburg, FB Organismische Biologie, Hellbrunnerstr. 34, 5020 Salzburg, Austria

## Abstract

**Background:**

The green crab *Carcinus maenas *is known for its high acclimation potential to varying environmental abiotic conditions. A high ability for ion and acid-base regulation is mainly based on an efficient regulation apparatus located in gill epithelia. However, at present it is neither known which ion transport proteins play a key role in the acid-base compensation response nor how gill epithelia respond to elevated seawater *p*CO_2 _as predicted for the future. In order to promote our understanding of the responses of green crab acid-base regulatory epithelia to high *p*CO_2_, Baltic Sea green crabs were exposed to a *p*CO_2 _of 400 Pa. Gills were screened for differentially expressed gene transcripts using a 4,462-feature microarray and quantitative real-time PCR.

**Results:**

Crabs responded mainly through fine scale adjustment of gene expression to elevated *p*CO_2_. However, 2% of all investigated transcripts were significantly regulated 1.3 to 2.2-fold upon one-week exposure to CO_2 _stress. Most of the genes known to code for proteins involved in osmo- and acid-base regulation, as well as cellular stress response, were were not impacted by elevated *p*CO_2_. However, after one week of exposure, significant changes were detected in a calcium-activated chloride channel, a hyperpolarization activated nucleotide-gated potassium channel, a tetraspanin, and an integrin. Furthermore, a putative syntaxin-binding protein, a protein of the transmembrane 9 superfamily, and a Cl^-^/HCO_3_^- ^exchanger of the SLC 4 family were differentially regulated. These genes were also affected in a previously published hypoosmotic acclimation response study.

**Conclusions:**

The moderate, but specific response of *C. maenas *gill gene expression indicates that (1) seawater acidification does not act as a strong stressor on the cellular level in gill epithelia; (2) the response to hypercapnia is to some degree comparable to a hypoosmotic acclimation response; (3) the specialization of each of the posterior gill arches might go beyond what has been demonstrated up to date; and (4) a re-configuration of gill epithelia might occur in response to hypercapnia.

## Background

With increasing atmospheric *p*CO_2_, a decrease in global surface ocean pH of between 0.4 to 0.8 units is predicted due to oceanic CO_2 _uptake [1,2]. Although large changes in atmospheric CO_2 _have been recorded throughout earth history, the current anthropogenic increase in *p*CO_2 _is much more rapid and severe than the cyclic changes of *p*CO_2 _during the last 20 million years [3]. The resulting changes in carbonate chemistry speciation, termed 'ocean acidification', may become a general stress factor modulating future marine communities by differentially influencing the fitness of marine species [4-7]. Many studies suggest complications for marine ectothermic metazoans in response to future ocean acidification: reduced growth and calcification rates, reduced rates of development, altered energy budgets, disturbed acid-base status, disturbed chemosensory function and even increased rates of mortality have been measured [4,7-14]. On the other hand, increases in growth and calcification rate were shown primarily in organisms that accumulate significant concentrations of bicarbonate in their body fluids, e.g. decapod crustaceans [12], cephalopods [15] and fish [16].

### Coping with ocean acidification

Biological impacts of acidification are strongly related to life history, genetic pre-disposition and physiological acclimation potential of the species in question. Elevated environmental *p*CO_2 _results in an increased extracellular CO_2 _partial pressure, as positive diffusion gradients of CO_2 _have to be maintained in order to excrete metabolic CO_2 _[5]. This can then lead to an acidification of extracellular fluids [17-19]. However, several active, high metabolic species with pH sensitive respiratory pigments regulate extracellular pH (pH_e_): active modulation of the extracellular carbonate system leads to bicarbonate accumulation and pH compensation while maintaining *p*CO_2 _values sufficiently high for diffusive CO_2 _flux out of the animal [fish: 20; crustaceans: 21; cephalopods: 22, 23]. In teleost fish, cephalopods, and decapod crustaceans, the majority of the acid-base relevant ion regulatory apparatus is located in gill epithelia. It is thought that net proton extrusion is primarily achieved via active (V-type H^+^-ATPase) and secondarily active ion transport molecules (e.g. sodium proton exchangers, NHE; sodium bicarbonate cotransporters, NBC), with a strong supporting role of carbonic anhydrases (CAs) and Na^+^/K^+^-ATPase [24].

### Carcinus maenas

In the case of the hyperosmoregulator *Carcinus maenas *(green or shore crab), the three posterior gills number 7-9 have been found to be involved in osmo- and acid-base regulation, while the anterior six gill pairs serve the demands of gas exchange [25-27]. Studies of Truchot [25] demonstrated that *C. maenas *actively and rapidly accumulates HCO_3_^- ^in its hemolymph during acute exposure to elevated seawater *p*CO_2 _(690 Pa). However, active adjustment of the extracellular carbonate system might not only be a short-term response in this species, but a persistent physiological response in order to maintain high extracellular pH (pHe). Despite efficient pHe regulatory control, other physiological aspects might be negatively impacted by hypercapnia and might show effects on the animal's performance only in the long run. Up to date, only few studies investigated long-term effects of acid-base disturbance in crustaceans on the physiological level. Potential reponses may include an altered calcification rate, as demonstrated for three crustacean species (the crab *Callinectes sapidus*, the shrimp *Penaeus plebejus *and lobster *Homarus americanus) *by Ries et al. [12], and other invertebrates, such as the cuttlefish *Sepia officinalis *[15]. Other adaptations to long-term hypercapnia may be similar to what has been observed as responses upon hypoxia, both factors being closely linked to each other. These might include energy conserving strategies (e.g. down-regulation of protein synthesis and modification of certain regulatory enzymes) as can be observed in response to hypoxia in mussels [28], fish [29] or reptiles [30] (reviewed by [31]).

On the molecular level, it is not known at present which ion transporters play a key role in the short- and the long-term response to hypercapnia in decapod crustaceans. Although models for the organization of the gill epithelia of euryhaline crabs have been postulated [32,33], the transporter inventory in decapod crustacean gill epithelia and their functional interactions are not fully understood at present. As *C. maenas *is exposed to regular short-term fluctuations in pH in its highly variable habitat in the western Baltic Sea [19], not only changes in gene expression levels may play a role in its acid-base compensation response. A compensatory stress response can as well take place on the post-transcriptional level e.g. through covalent modification of certain regulatory enzymes and their activity through phosphorylation-dephosphorylation reactions. This has been shown e.g. for the enzyme phosphofructokinase, the key enzyme in controlling anaerobic carbon flow in glycolysis in mussels in response to hypoxia [34], and fish [35]. A post-translational alteration has also been hypothetized for the enzyme carbonic anhydrase in the blue crab *Callinectes sapidus*, a close relative of *C. maenas *[36].

### Aim of the study

In order to promote our understanding of ion and acid-base regulatory processes, green crabs from the Baltic Sea were exposed to control (39 Pa) and elevated (400 Pa) *p*CO_2 _(100 Pa = 987 μatm). The experimental conditions in the laboratory were chosen to mimick the parameters in the crabs' natural environment, the brackish water system of the western Baltic Sea. The chosen experimental level of *p*CO_2 _enrichment (400 Pa) represents a stress scenario that could occur in the natural habitat within the next 100 years: pH in Kiel Fjord can reach values as low as 7.5 (corresponding *p*CO_2 _= 230 Pa) in surface waters during summer and autumn. Future changes in *p*CO_2 _will be more severe in this habitat than in the average surface ocean [19]. As a strong extracellular acid-base reaction has been observed in *C. maenas *exposed to hypercapnia [25], we hypothesize that the ion regulatory transcriptome of *C. maenas *will respond to hypercapnia with a short- and long-term adjustment of expression of important ion- and acid-base transporter candidates (e.g. Na^+^/K^+^-ATPase, Na^+^/H^+ ^exchanger (NHE), V - type H^+^-ATPases, Cl^-^/HCO_3_^- ^exchangers and carbonic anhydrases; [33]). As little is known about candidate genes for acid-base regulation in crustaceans, we chose to utilize a microarray approach to screen for previously unrecognized candidates. Therefore, gene expression profiles of gills of short-term (3 and 7 days) exposed crabs were investigated using a 4,462-feature microarray assay recently developed by Towle et al. [37]. Expression levels of distinct candidate genes were further investigated using quantitative real-time PCR analysis following long-term exposure to hypercapnia (11 weeks). Expression profiles obtained in our study were compared to those obtained in a recent hyposmotic study, in which gene expression changes after transfer of *C. maenas *from a salinity of 32 to 15 was investigated using the same microarray [37]. A second focus of interest was placed on genes known to be in involved in the cellular stress-response (e.g. heat-shock proteins, superoxide dismutase, glutathione reductase [38]).

This is the first study that elucidates the impact elevated seawater *p*CO_2 _has on the gill transcriptome of a decapod crustacean.

## Methods

### Animals, exposure, and tissue sampling

#### Short-term CO_2 _experiments

Green crabs (*Carcinus maenas*) were caught in traps in April 2009 in Kiel Fjord (Baltic Sea) at the IFM-GEOMAR pier (westshore building) at 3-4 m depth (54°19.8'N; 10°9.0'E). In order to acclimate the animals to laboratory conditions, they were kept in a flow-through tank (H30 × W30 × L50 cm) in a climate chamber at IFM-GEOMAR with ambient aerated Baltic Sea brackish water at 13°C for 3 to 7 days. Experimental animals were fed *ad libitum *with mussels (*Mytilus edulis*) during this time. Female crabs (n = 24, 6 animals per CO_2 _treatment and day) with a mean carapace width of 4.6 ± 0.2 cm were then chosen for the short-term experiment and two each were transferred to 20 L tanks of a flow-through seawater CO_2 _manipulation system (flow rate = 100 mL min^-1^) that was supplied with ambient Baltic Sea brackish water of a salinity of 14 - 15 (Practical salinity scale, as it will be used throughout the script). Temperature was held constant in the storage tank by a thermostat set to 13°C. A light-dark cycle of 12:12 h was established. The system design was identical to that presented in Thomsen et al. [19].

After 24 hours of acclimation in the experimental system resembling the natural parameters encountered in Kiel Bight (S = 14.9, T = 12.9 ± 0.1°C), crabs were exposed to control (53 Pa, pH = 8.11 ± 0.04) and elevated (440 Pa, pH = 7.24 ± 0.03) *p*CO_2 _for 3 or 7 days. CO_2 _was provided by a central automatic CO_2_-mixing-facility (Linde Gas, HTK Hamburg, Germany). Animals were fed *ad libitum *with crushed mussels (*Mytilus edulis*).

After 3 days, one of the two animals in each tank was removed for analysis, anaesthetized on ice and then killed by destroying its ventral ganglion and removal of the carapace. Left gills #7 and #9 were carefully removed with forceps and immediately stored in RNAlater^® ^(Ambion, #AM7021) until microarray analysis at the Mount Desert Island Laboratory, Salisbury Cove, Maine/USA, or quantitative real-time PCR (qRT-PCR at the IFM-GEOMAR, Kiel. The procedure was repeated for the second animal of each tank after 7 days of incubation.

For qRT-PCR on gills of short-term exposed animals, a repeated incubation of *Carcinus maenas *specimens was performed in May 2010. Again, Baltic Sea green crabs were caught in traps in Kiel Fjord at the IFM-GEOMAR pier. Females had a mean carapace width of 4.8 ± 0.6 cm. System parameters in the acclimation phase were S = 13.4 and T = 10.8 ± 0.1°C. The animals (n = 12, 6 animals per CO_2 _treatment and day) were then exposed to seawater of pH 7.99 ± 0.02 (68 Pa CO_2_) and pH 7.28 ± 0.05 (361 Pa CO_2_) as described above. Gills were isolated only after 7 days in this experiment.

#### Abiotic seawater parameters

To evaluate the carbonate system parameters, water samples (500 mL) were taken of 4 randomly chosen tanks each for day 3 and day 7 of the short-term hypercapnia experiment in 2009. For the second short-term exposure in 2010, water samples of 4 control and 4 experimental aquaria were taken only on day 7. Total dissolved inorganic carbon (*C*_T _= [CO_2_*] + [HCO_3_^-^] + [CO_3_^2-^]) was measured according to Dickson and Millero [39]. In case of the second 1 week exposure in 2010, *C*_T _was measured using an AIRICA CT analyzer (Marianda GmbH, Kiel, Germany). In all experiments, salinity, pH and temperature were assessed daily using a pH- and salinometer (WTW 340i pH-analyzer/WTW SenTix 81-measuring chain, WTW cond 315i/WTW TETRACON 325-measuring chain).

Seawater total alkalinity (*A*_T_) and *p*CO_2 _was calculated from pH and *C*_T _using CO2SYS software [40] and the appropriate parameter and constants (dissociation constants K1 and K2 according to Roy et al. [41], KHSO_4 _dissociation constant after Dickson [42], NBS scale [mol/kg H_2_O]; table 1).

**Table 1:** Carbonate system parameters of all hypercapnia experiments with *Carcinus maenas*

	1 week experiment 2009		1 week experiment 2010		11 week experiment 2009	
**Parameter**	**control**	**experiment**	**control**	**experiment**	**control**	**experiment**

C*_T _*[μmol/kg] (measured)	2025 ± 29	2242 ± 37	1879 ± 7	2047 ± 10	1999 ± 18	2209 ± 24

A*_T _*[μmol/kg] (calculated)	2098 ± 29	2062 ± 26	1904 ± 5	1887 ± 9	2046 ± 19	2061 ± 20

pH (measured)	8.12 ± 0.02	7.24 ± 0.02	8.00 ± 0.02	7.28 ± 0.01	8.06 ± 0.01	7.36 ± 0.01

S (measured)	15.35 ± 0.06	15.30 ± 0.00	13.40 ± 0.02	13.4 ± 0.02	14.80 ± 0.13	14.80 ± 0.13

T (measured) [°C]	12.90 ± 0.16	12.98 ± 0.05	10.87 ± 0.02	10.77 ± 0.13	12.90 ± 0.07	12.90 ± 0.07

*p*CO_2 _(calculated) [Pa]	53 ± 2	440 ± 25	68 ± 3	361 ± 9	40 ± 5	230 ± 21

Measured parameters of the carbonate system were assessed daily during the acclimation phase of 1 or 11 weeks, while A_T _and *p*CO_2 _were calculated applying the measured parameters and CO2SYS software (see Material and Methods for details). Under experimental conditions (elevated *p*CO_2_), a drop in pH of 0.7 - 0.9 units could be observed. The carbonate system remained stable during the acclimation phase in both, short- (1 week) and long-term (11 weeks) experiments. C_T _= total dissolved inorganic carbon, A_T _= total alkalinity, S = salinity, T = temperature, *p*CO_2 _= partial pressure of CO_2_. Values are given as mean with standard deviation.

#### Long-term CO_2 _experiments

**Figure 1:**
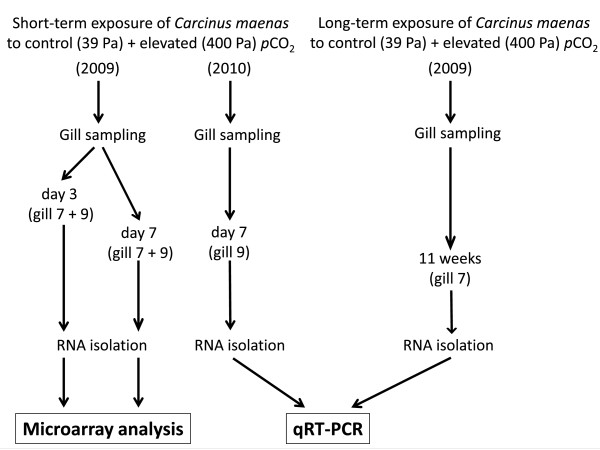
**Overview over the experiments performed on *Carcinus maenas *specimen of the Baltic Sea**. A short-term incubation experiment was conducted in April 2009 and microarray analysis was performed on isolated RNA of gills of these animals exposed to control (nominal 39 Pa) and elevated (nominal 400 Pa) *p*CO_2 _for 3 and 7 days. The experiment was repeated in 2010 and this time, RNA was isolated for quantitiative real-time polymerase chain reaction (qRT-PCR) of gill 9 after 7 days. In parallel, a long-term experiment was performed on Baltic Sea *C. maenas *in 2009 by Appelhans et al. (under review), and RNA of gill 7 was isolated and analysed by qRT-PCR.

Green crabs (*Carcinus maenas*) for the long-term experiment were part of a study by Appelhans et al. (under review) that was conducted in parallel. Experimental animals for this long-term study were caught in traps in April 2009 in Kiel Fjord (Baltic Sea) at the IFM-GEOMAR pier (westshore building) in 3 to 4 m water depth (54°19.8'N; 10°9.0'E). Animals with a carapace width of 4.5 ± 0.5 cm were chosen irrespectively of their sex (n = 9 animals per CO_2 _treatment). Crabs were exposed to control (40 Pa, pH = 8.06 ± 0.01) and elevated *p*CO_2 _(230 Pa, pH = 7.36 ± 0.01). Animals were fed with 2 mussels (size = 2.50 ± 0.35 cm) three times a week. To evaluate the carbonate system parameters, water samples of three aquaria per treatment level were taken at the beginning, the intermediate phase and at the end of the experiment, respectively. Analysis of *C*_T _was coulometric (SOMMA System autoanalyser, Marianda GmbH, Kiel, Germany) and *A*_T _analyzed via potentiometric titration (VINDTA autoanalyser, Marianda; table 1). An overview over the different experiments and sampling points is depicted in Figure 1.

### Microarray experiment

#### RNA extraction

RNA from the first short term experiment (2009) was extracted from whole left gills #7 and #9 (control *p*CO_2_, n = 6; elevated *p*CO_2_, n = 6) using the RNeasy Midi-Kit from Qiagen (#75144). Gills were homogenized with an OMNI international TH/G7-195STW for 50 s in 4 mL buffer RLT from the Qiagen kit. Extraction then followed the manufacturer's description. RNA quality was monitored by electrophoresis via the Agilent 2100 Bioanalyzer system. Each RNA sample was then reversely transcribed into cDNA and in parallel fluorescence-labeled using the SuperScript™ Plus Direct cDNA Labeling System from Invitrogen (#L1015-06), following the provided protocol. Using both anchored oligo(dT)20 and random hexamer primers included in the kit, control cDNA was labeled with AlexaFluor555 (green fluorescent, equivalent to Cy™3; excitation: 555 nm, emission: 565 nm) while cDNA of the CO_2_-treatment samples was labeled with AlexaFluor647 (red fluorescent, equivalent to Cy™5; excitation: 650 nm, emission: 670 nm).

#### Microarray assay for Carcinus maenas

The *C. maenas *microarray was developed by D. W. Towle et al. and prepared as described in Towle et al. [37]. The annotation of the respective contigs included in the microarray, based on a *Carcinus maneas *EST library (deposited at http://www.ncbi.nlm.nih.gov), was performed by Towle et al. [37] using BLASTX analysis on a local TimeLogic DeCypher server (Active Motif, Inc.). The most informative BLASTX hit for each contig sequence was selected manually from the ten highest scoring hits identified by the programm. In order to improve the information on each included gene, a re-annotation of the contigs has been performed in 2009 by the authors of this study and compared to the existing annotation (Additional File 1 Table S1). A sequence blast with the online portal Blast2GO [43] was performed to improve the quality of the annotations. Additionally, a mapping and substance Gene Ontology (GO) annotation analysis was performed to amend information of biological functions [44], including: (1) Enzyme Commission number (EC), (2) InterPro scan, (3) annotation augmentation through the Second Layer concept (ANNEX) and (4) summarizing annotation results by creating a subset of the gene ontology vocabulary encompassing key ontological terms (GOslim). Additionally, direct NCBI blasts were performed for selected genes of special interest.

#### Microarray analysis of the short-term samples (3 and 7 days)

Hybridization of the microarray slides was performed using the MAUI Hybridization system (MAUI Mixer SC Hybridization Chamber mixers, Biomicro Cat. no. 02A00830) using the Pronto!Universal Hybridization Kit. Slides were scanned in an Axon GenePix 4000B dual wavelength scanner using the GenePix 6.0 software.

Initial within-slide normalization (lowess-normalization) was performed by the Acuity 4.0 software (Input parameter: Smoothing = 0.19 (chosen by graphical/visual evaluation), Iterations = 3 (default), Delta = 0.01 (default)). For further processing and statistical analysis, data was then exported to Excel. Filtering of the data set started with excluding low-quality features according to the "flagging" performed in Acuity 4.0. Genes were defined "absent" and excluded from further analysis, when the fluorescence intensity of one or both channels was less than 30% higher than the background fluorescence intensity. Also, transcripts with a variance of more than 20% in the 4 technical replicates included in each slide were not included in the data set. Gene expression was calculated as the log_2 _of the ratio of the fluorescence intensity of the CO_2_-treatment cDNA to the fluorescence intensity of the control cDNA (log_2_-ratio = F635/F532) and as the median and median deviation (MD) of each 6 replicate microarray slides per block (gill7day3, gill7day7, gill9day3, gill9day7).

### Quantitative polymerase chain reaction (qRT-PCR)

#### RNA extraction

RNA from whole left gills #9 (only day 7, control CO_2_, n = 6; elevated CO_2_, n = 6) from the second 1 week experiment (2010) and from whole left gills #7 (control CO_2_, n = 9; elevated CO_2_, n = 9) of the 11 week experiment was extracted using the RNeasy Midi-Kit from Qiagen (#75144). Gills were first homogenized with an OMNI international TH/G7-195STW for 50 s in 4 mL buffer RLT from the kit. Extraction then followed the manufacturer's description.

#### Primers

A number of transcripts shown to undergo changes in the 1 week microarray experiment and/or known to be involved in acid-base regulation were selected for analysis by qRT-PCR, according to their relevant function and statistical significance. Primers were designed according to the database previously generated by D.W.Towle [37], using PrimerExpress software (Version 2.0, Applied Biosystems). Primer quality and specificity was tested by blasting with the EST library for *Carcinus maenas *from NCBI. Forward and reverse primers were generated with the following parameters: GC content 30-80%, primer length 9-40 bp (opt. 20 bp), Amplicon melting temperature 0-85°C, Amplicon length 70-150 bp (Additional File 1 Table 2). Performance and efficiency of each primer pair was tested in triplets in a cDNA dilution series (1:40, 1:80, 1:160, 1:320, 1:640). Performance was evaluated as suitable if the R^2 ^of the linear regression of the dilution series was > 0.98 and no background noise (unspecific binding) was detected. A melting curve analysis was performed for each reaction to ensure a single PCR amplicon. Efficiency was calculated as follows:

**Table 2:** Enrichment analysis in the short-term hypercapnia microarray study on Carcinus maenas

Test-set	GO	name	FDR	Over-/Under- representation
down day 3	GO:0050794	regulation of cellular process	0.00	under

down day 3	GO:0007165	signal transduction	0.00	under

down day 3	GO:0023060	signal transmission	0.00	under

down day 3	GO:0023046	signaling process	0.00	under

down day 3	GO:0003824	catalytic activity	0.00	under

down day 3	GO:0050789	regulation of biological process	0.04	under

up day 3	no enrichment	-	-	-

down day 7	no enrichment	-	-	-

up day 7	no enrichment	-	-	-

up gill 7	GO:0007010	cytoskeleton organization	0.05	under

down gill 7	GO:0005623	cell	0.04	over

up gill 9	GO:0005198	structural molecule activity	0.03	over

down gill 9	no enrichment	-	-	-

Results of the enrichment analysis (Fisher's Exact Test with multiple testing correction; FDR < 0.05) as implemented in Blast2GO [43]. Tested were subsets of all significantly regulated transcripts as identified by sign test (up- and down-regulated separately for each gill and day or each combination of both). Reference-set in each analysis was the complete microarray sequence Blast2GO database generated from the contig list. GO = Gene Ontology term, name = GO name of the functional group, FDR = false discovery rate for correction of multiple testing, Over-/Underrepresentation = a GO term is considered over-/under-represented if it appears significantly more often/less often in the reference-set than it does in the test-set.

E = 10 ^(-1/S)^, with S being the slope of the linear regression of the dilution series. Only primer pairs with an efficiency between 1.9 - 2.1 were chosen for analysis.

#### qRT-PCR

Following DNase digestion of 2 μg total RNA with the DNAfree™ Kit from Ambion (#AM1906), 0.4 μg DNAfree RNA was then transcribed into cDNA using the High Capacity cDNA RT Kit from Applied Biosystems (Part no. 4368814). Real-time PCR was performed on 80-fold diluted cDNA in 96-well plates using the FastSYBR^®^Green Master Mix (Part no. 4385612) and the StepOnePlus™ Real-Time PCR System from Applied Biosystems (PCR-conditions: 20 s at 95°C, 40 cycles of 5 s at 95°C and 30 s at 62°C). On each plate, one control cDNA and one experimental cDNA were analyzed with the same primer pairs, as well as the respective DNAfree RNA for each of the two samples as control for DNA contamination, and arginine kinase as housekeeping gene to eliminate within-plate variation. Arginine kinase was chosen as housekeeping gene according to [36] and was tested to be appropriate (stability value = 0.007) with NormFinder ([45], available at http://www.mdl.dk/publicationsnormfinder.htm). Only those samples were included in the analysis that showed no or low background in the DNAfree RNA control compared to the cDNA samples (difference in cycles > 9 ≈ 0.3%). Gene expression was calculated based on the C_T_-threshold. First, absolute quantities Q_X _of all genes were calculated as

$Q_{X\left(gene\right)}={\left(E\right)}^{C_{T}}$, followed by the calculation of normalized quantities

$Q_{N\left(gene\right)}=\frac{Q_{X\left(gene\right)}}{Q_{X\left(HKgene\right)}}$, with Q_X (HK gene) _being the absolute quantity of the housekeeping gene arginine kinase. For matters of consistency with the microarray data, gene expression resulting from qRT-PCR analysis was also calculated as the log_2_-ratio of normalized gene quantity in elevated *p*CO_2 _treated animals to normalized gene quantity in control *p*CO_2 _animals.

### Statistics

Because of the high biological variance within replicate samples, non-parametric statistical tests were chosen. To identify significant changes in gene expression, a two-sided sign test with α = 0.05 was performed on the fluorescence intensities of the filtered and lowess-normalized data set for each of the four experimental blocks (gill7day3, gill7day7, gill9day3, gill9day7). For each individual transcript in each block, the fluorescence intensities for the F635-dye, representing the animals being exposed to elevated *p*CO_2_, were tested against the control animals (F532-dye). The test only allowed for including those transcripts that were represented by 5 or 6 replicates.

Sign test was also applied on the experimental vs. control normalized gene quantities of the qRT-PCR.

In order to detect differences between gills and days of the short-term exposed animals, Wilcoxon's matched pairs test as implemented in STATISTICA 8 was applied with α = 0.05. Prior to testing, microarray values were transformed into a matrix with 1 = significantly up-regulated, -1 = significantly down-regulated, 0 = not significantly regulated transcript. Data of each sampling day and gill were then tested against each other.

Additionally, intensity values for both fluorescence dyes of the processed microarray data (lowess-normalized and filtered as described above) was read into the R software environment (version 2.13.1, http://www.R-project.org), using the R/maanova package (version 2.22.0, [46]). As the package does not allow for missing data, only transcripts represented by all 6 replicates could be tested by this method. A linear mixed model

yijk=μ+CO2i+Gj+Dk+eijk

was fitted to the data, with *yijk *as the normalized-transformed gene expression, μ as the group mean, *CO2i *as the effect of the CO_2 _level, *Gj *as the effect of ith gill, *Dk *as the effect of jth day, and *eijk *as the sample effect (random error). The significance of effects of the fixed factors "CO_2 _treatment level", "gill" and "day" on the differential expression of genes was tested by appropriate contrasts in F-tests between groups in a multifactorial ANOVA design. The F-tests were calculated with the James-Stein shrinkage estimate (Fs), incorporating shrinkage estimates of variance components [47]. P values were calculated by performing 1,000 permutations of samples to break their association to expression values, then corrected for multiple comparisons by false discovery rate transformation (FDR), using the qvalue package (jsFDR, version 1.26.0 [48]) with a 5% FDR cutoff.

To identify overall affected pathways, enrichment analysis (FDR < 0.05) was performed using Fisher's exact test as implemented in Blast2GO [43]. All significantly regulated genes identified by sign test, divided into subsets for each gill and day, were used as test-sets and tested against a complete microarray sequence Blast2GO database generated from the contig list. It has to be considered that *Carcinus maenas *is a non-model organism and that therefore the annotation of gene functions/Gene ontology (GO) terms is not comprehensive.

## Results

### General findings of the microarray analysis

**Figure 2:**
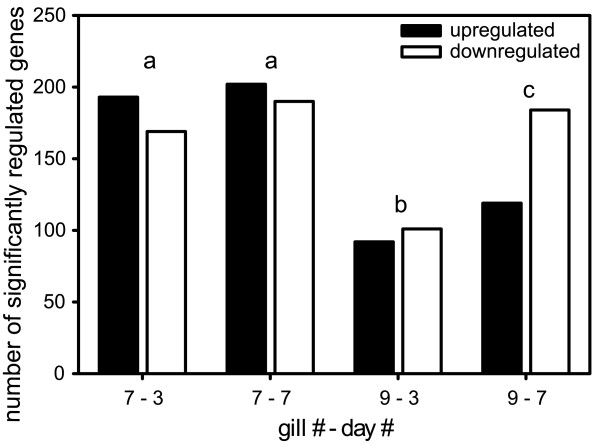
**Differentiation of the sum of all significantly regulated transcripts for each gill and day of *Carcinus maenas *upon short-term exposure to hypercapnia**. For each of the experimental blocks of the short-term hypercapnia experiment (gill7day3, gill7day7, gill9day3 and gill9day7), up- and down- regulated transcripts were counted and represented as bars. Additionally, a matrix was generated for all significantly regulated transcripts (sign test) for each block with 1 = significantly up-regulated, 0 = not significantly regulated, -1 = significantly down-regulated. Statistical comparison of the four experimental blocks was done applying Wilcoxon's matched pairs test on the matrices. No significant difference was found in gill 7 comparing the two sampling points, while gill 9 on day 3 differed significantly compared to gill 9 on day 7 (p < 0.05). Additionally, gill 9 in general differed significantly in comparison to gill 7 on both days (p < 0.05). Different letters a, b, c denote these significant difference.

Using variance-based analysis utilizing the R/maanova package in R, we only identified the factor "CO_2_" to have a significant effect on gene expression, while factors "Gill" and "Day" did not significantly influence expression patterns. 956 out of 3,720 tested transcripts were found to be differentially expressed between the two CO_2 _levels. This accounts for 26% and is comparable to the result of the sign test (24%, see below). 378 (40%) of the transcripts identified to be differentially expressed for the factor CO_2 _in the variance-base approach were also identified as significantly regulated by sign test, including 11 (65%) of the transcripts of special interest as presented in table 3. The sign test identified as many as 1,056 (24%) out of the 4,462 transcripts on the microarray to be significantly up- or down-regulated in at least one gill or at one sampling time point during the short-term response to elevated seawater *p*CO_2 _(p ≤ 0.05; Figure 2; Additional File 1 Table S3). Significant up-regulation was observed in 541, down-regulation in 502 genes (13 genes showed a mixed regulation pattern). However, magnitudes of change were generally low: expression of only 84 genes of the significantly regulated transcripts (sign test, accounting for 2% of the whole microarray and 8% of the significantly altered transcripts) were modulated by a log_2_-ratio > |0.200| (sign test; equivalent to a > 1.3-fold change). F-test identified 16 additional transcripts with a log_2_-ratio > |0.200|.

**Table 3:** Regulation of transcripts of special interest in the short-term hypercapnia study on *Carcinus maenas*

			CO_2 _experiments 3 + 7 days				CO_2 _experiments 11 weeks	Salinity experiments 7 days
			[MA; med. log2-ratio ± MD]				[qPCR; med. log2-ratio ± MD]	[MA; mean log2-ratio ± SD]
rank	**ACC. no**.	Name	gill 7 - day 3	gill 9 -day 3	gill 7 - day 7	gill 9 -day 7	gill 7 - 10 weeks	gill 8 - day 7
1	**DV944570.1**	senescence-associated protein	-0.12 ± 0.50	-0.03 ± 0.03	-1.16* ± 0.64	-0.07 ± 0.25	0.69 ± 0.57	-0.06 ± 0.03

2	*DW250369.1*	pg1 protein	1.01 ± 0.61	0.60 ± 1.14	-0.34 ± 0.49	-0.29 ± 0.56	-	1.644 ± 0.414

3	**DN161290.1**	unknown (putative Syntaxin binding protein 2)	0.32 ± 0.32	0.42 ± 0.10	0.88* ± 0.10	0.67 ± 0.31	0.49 ± 0.66	2.86 ± 0.02

4	**DN796131.1**	23S ribosomal RNA gene (a)	-1.20 ± 0.92	-0.25 ± 0.18	-1.14 ± 1.16	-0.87* ± 0.46	-	0.36 ± 0.34

5	**DW584691.1**	calcium-activated chloride channel	0.25 ± 0.40	0.81* ± 0.34	-0.09 ± 0.30	0.16 ± 0.31	1.12 ± 0.88	-

6	**DN635040.1**	23S ribosomal RNA gene (b)	-0.16 ± 1.42	0.09 ± 0.64	-0.80* ± 0.22	-0.27 ± 0.49	-	1.29 ± 0.16

7	*DV944193.1*	rRNA intron-encoded homing endonuclease	0.54 ± 1.00	0.80 ± 0.81	0.06 ± 0.22	0.59 ± 0.41	-	1.073 ± 0.38

8	**DW249535.1**	unknown	-0.12 ± 1.43	0.04 ± 0.55	-0.71* ± 0.62	-0.41 ± 0.65	-	0.99 ± 0.29

9	**DY656451.1**	unknown	-0.15 ± 0.23	0.05 ± 0.26	0.57* ± 0.09	0.04 ± 0.27	-	-0.15 ± 0.30

10	**DW251049.1**	unknown	-0.35 ± 0.24	-0.53* ± 0.28	-0.05 ± 0.22	-0.10 ± 0.19	-	-0.86 ± 0.23

11	**DN551127.1**	unknown	0.54 ± 0.25	0.26 ± 0.14	0.48* ± 0.31	0.37 ± 0.48	-	2.34 ± 0.07

12	**DW250828.1**	thymosin-repeated protein 1	-0.39 ± 0.21	-0.46* ± 0.14	0.13 ± 0.21	0.19 ± 0.21	-	-0.05 ± 0.06

13	**DN202592.1**	multispanning membrane protein (transmembrane 9 superfamily protein member 4)	0.35 ± 0.23	0.21 ± 0.14	0.46* ± 0.11	0.36 ± 0.34	-	2.15 ± 0.08

14	**DW250260.1**	hyperpolarization activated cyclic nucleotide-gated potassium channel-2	-0.26 ± 0.30	-0.14 ± 0.08	-0.46* ± 0.24	-0.14* ± 0.08	-0.13 ± 0.26	-0.11 ± 0.09

18	**DV944030.1**	tetraspanin 3 (Transmembrane super family 4 member 8)	-0.22 ± 0.11	-0.41* ± 0.05	0.01 ± 0.27	0.09 ± 0.03	-	-0.13 ± 0.06

25	**DY307809.1**	potential integrin alpha-7	-0.12 ± 0.36	0.26 ± 0.21	-0.35* ± 0.28	-0.03 ± 0.26	-	-

29	**DV642643.1**	kazal-type proteinase inhibitor	0.25* ± 0.17	0.21* ± 0.12	0.33* ± 0.20	0.24* ± 0.15	0.12 ± 0.84	1.04 ± 0.11

31	**DN635395.1**	unknown	-0.17* ± 0.04	-0.14* ± 0.04	-0.32* ± 0.06	-0.13* ± 0.06	0.25 ± 0.68	-0.11 ± 0.11

63	**DV943872.1**	predicted ATPase	-0.34 ± 0.33	-0.24 ± 0.10	-0.58 ± 0.39	-0.23* ± 0.09	-	-0.65 ± 0.23

88	**CX994129.1**	anion/bicarbonate transporter family member (SLC family 4, member 1)	-0.31 ± 0.11	-0.20* ± 0.09	-0.22 ± 0.07	-0.22 ± 0.11	0.18 ± 0.54	0.41 ± 0.11

-	**DN739347.1**	glycosyl-phosphatidylinositol-linked carbonic anhydrase VII	-0.09 ± 0.05	-0.06 ± 0.06	-0.13* ± 0.07	-0.08* ± 0.03	0.40 ± 0.35	-0.92 ± 0.07

-	**DN551450.1**	glutathione peroxidase	0.02 ± 0.06	0 ± 0.05	0.03* ± 0.02	-0.03 ± 0.05	-0.90 ± 0.24	-0.81 ± 0.14

-	DY656042.1	vacuolar-type H+-ATPase subunit A	-0.05 ± 0.37	0.11 ± 0.09	-0.11 ± 0.09	0.07 ± 0.06	0.45 ± 0.65	-0.17 ± 0.27

-	DY657461.1	v-type proton ATPase subunit E	0.02 ± 0.03	-0.02 ± 0.04	0 ± 0.03	-0.02 ± 0.03	0.30 ± 0.39	-0.12 ± 0.17

-	DW251095.1	cytoplasmic carbonic anhydrase	0.07 ± 0.01	-0.03 ± 0.06	0 ± 0.07	0.01 ± 0.01	-0.12 ± 1.20	1.78 ± 0.16

-	DN202373.1	anion/bicarbonate transporter family member (SLC family 4, member 11)	-0.02 ± 0.03	0.02 ± 0.06	-0.02 ± 0.10	0.01 ± 0.02	0.02 ± 0.64	-1.29 ± 0.47

-	DN796352.1	K+/Cl- symporter (solute carrier family 12, member 6)	-0.04 ± 0.04	0.22 ± 0.17	0.01 ± 0.04	-0.07 ± 0.07	0.91 ± 0.30	0.25 ± 0.23

-	DV111894.1	Na+/K+/2Cl- cotransporter	0.05 ± 0.05	0.01 ± 0.05	0 ± 0.06	0 ± 0.10	0.62 ± 0.57	-0.20 ± 0.09

-	DW250552.1	Na+/K+ ATPase alpha subunit	0.04 ± 0.05	0.02 ± 0.05	0.05 ± 0.10	0.03 ± 0.16	0 ± 1.11	1.38 ± 0.11

-	DY308103.1	Na+/H+ exchanger (NHE 3)	-	-	-	-	0.27 ± 0.03	-0.13 ± 0.14

Included are the 14 most strongly regulated transcripts (as identified by sign test and F-test), as well as several transcripts which have a potential function during structural modifications or in ion- and acid/base regulation. Transcripts are ordered according to the rank in absolute regulation. Accession numbers (ACC. no.) refer to the ESTs generated for the *C. maenas *microarray by Towle et al. [37] in the database GenBank (NCBI). Rank 1 = highest regulation; bold and underlined = significant regulation relative to the control identified by sign test; bold = identified to be differentially expressed by both tests; italic = identified to be differentially expressed only by F-test; "-" = a rank was not assigned in case the regulation was less than log_2_-ratio = |0.20|.

Enrichment analysis demonstrated that "structural molecule activity" (Gene ontology term (GO):0005198) was over-represented in all significantly up-regulated transcripts of gill 9 (table 3). Additionally, "cell" (GO:0005623) was over-represented in all significantly down-regulated transcripts of gill 7. No other over-representation of any GO-term was identified. However, 7 GO-terms were under-represented for different subsets of all significant regulated transcripts in the same analysis.

#### Regulation of specific groups

##### Stress response

Out of 137 identified transcripts associated with the cellular stress response (according to [38]), only 30 genes were significantly regulated (sign test with p < 0.05), albeit less than 1.1-fold (20 up-, 9 down-regulated transcripts; Additional File 1 Table S4). Variance-based linear modeling identified 23 genes to be differentially expressed, including 5 that were identified also by sign test. Expression of only 5 out of 26 heat-shock proteins (HSPs) was significantly altered (2 up-, 3 down-regulated transcripts) in sign test, while 5 different HSPs were identified by variance-based analysis. Additionally, stress-associated GO-terms were not found to be significantly enriched in the gills of short-term exposed animals (Fisher's exact test). The expression level of glutathione peroxidase [GenBank: DN551450.1] was also investigated in the long-term exposed animals (qRT-PCR analysis) and did not show a significant change.

##### Structural modification

In total, 77 transcripts were associated with GO: 0005198 (structural molecule activity). Significant up-regulation of 13 genes (sign test, 10 of them also identified by F-test) from this group resulted in the over-representation of this GO-term in gill 9 (Additional File 1 Table S5). These up-regulated genes are eight ribosomal proteins, a cuticular protein, two alpha-tubulins, a keratin-associated protein and a vitelline membrane outer layer protein. The identification of a 1.4-fold up-regulated transcript, encoding for a multispanning endomembrane protein of the transmembrane 9 superfamily (protein member 4 = TM9SF4, [GenBank:DN202592.1]), also suggests structural modification of gill membranes. It was up-regulated in both gills on both sampling days (sign test with p < 0.05). This transcript was also identified to be up-regulated in the low salinity study [37]. Additionally, two other transmembrane proteins were identified to be strongly down-regulated (sign test with p < 0.05): a tetraspanin-like 8 protein ([GenBank:DV944030.1], included in the GO-class "cell") was found to be strongly down-regulated during short-term hypercapnia (1.3-fold) on day 3 and slightly during low salinity exposure. A putative Integrin-alpha-7 [GenBank:DY307809.1] was identified to be strongly down-regulated (1.3-fold, sign test with p < 0.05) on day 7 (table 3, Additional File 1 Table S5).

##### Specialization of the gills

**Figure 3:**
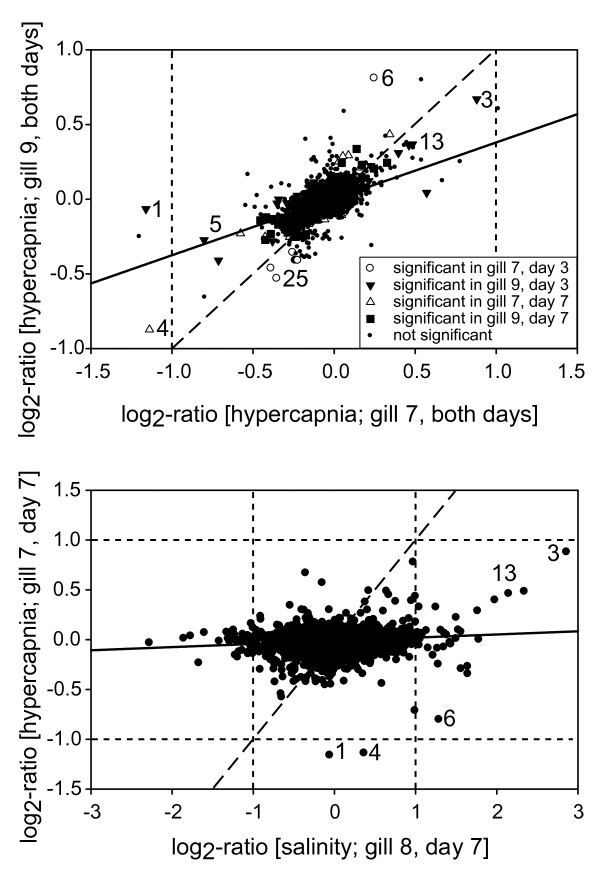
**Comparison of effects in transcript regulation in gills of *Carcinus maenas *upon short-term exposure to hypercapnia and low salinity**. (A) Regression analysis of gill 9 vs. gill 7 (both days) in the short-term hypercapnia experiment and (B) of gill 7/day 7 (short-term hypercapnia experiment, this study) vs. gill 8/day 7 (salinity dilution experiment by Towle et al. [37]). The long-dashed black line represents a theoretical regression ratio of 1:1 (equal influence of both factors), while the black line shows the actual regression. Transcripts outside the short-dashed horizontal and vertical lines are more than 2-fold regulated. Please note the different scaling in (A) and (B). Numbers are according to the gene's rank in table 3, indicating transcripts discussed in further detail in the manuscript. (1) senescence-associated protein [GenBank:DV944570.1], (3) putative Syntaxin binding protein 2 [GenBank:DN161290.1], (4) 23S ribosomal RNA gene (a) [GenBank:DN796131.1], (5) calcium-activated chloride channel [GenBank:DW584691.1], (6) 23S ribosomal RNA gene (b) [GenBank:DN635040.1], (13) multispanning membrane protein (transmembrane 9 superfamily protein member 4 [GenBank:DN202592.1], (25) potential integrin alpha-7 [GenBank:DY307809.1].

Although no effect of the factor "gill" on individual gene expression was identified by variance-based linear modeling, Wilcoxon's matched pairs test identified a significant difference in the overall gene expression patterns in the different experimental blocks (gill7day3, gill7day7, gill9day3, gill9day7; p < 0.05; Figure 2, Figure 3A), based on total numbers of up-, down-, or not regulated transcripts. Fewer genes were significantly regulated in gill 9 after both 3 and 7 days of exposure than in gill 7 (sign test). In total, 756 genes were significantly regulated in gill 7, whereas only 510 in gill 9. Upon hypercapnia stress, gill 7 in general shows stronger responses to hypercapnia than gill 9. For example, the strongest overall significant change in gene expression was observed in gill 7 of a senescence-associated protein (sign test and F-test with p < 0.05, [GenBank:DV944570.1]), a gene that was not altered in gill 9, regarding both, day 3 and 7. On the other hand, gene expression of a calcium-activated chloride channel [GenBank:DW584691.1] was only significantly and strongly affected in gill 9 on both days of the short-term exposure (sign test and F-test with p < 0.05). Additionally, according to the enrichment analysis, structural changes in gill epithelia are linked mainly to gill 9.

##### Comparison with gene expression patterns in response to low salinity

**Figure 4:**
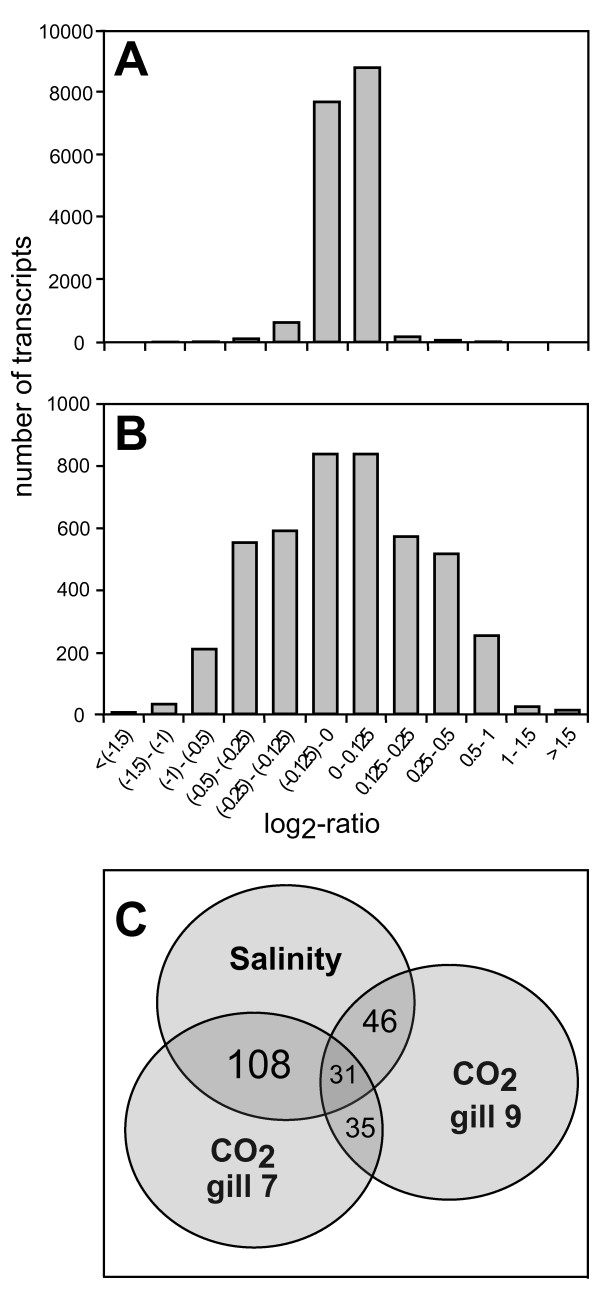
**Comparison of regulation frequencies in *Carcinus maenas *gills in salinity and CO_2 _experiments**. (A) Frequency distribution of all gene expression levels in gill 7 and 9 on day 7 as response to short-term hypercapnia (this study). (B) Frequency distribution of all gene expression levels in gill 8 on day 7 as response to low salinity (study of Towle et al. [37]). Please note the different scaling of the y-axis in (A) and (B). Exposure to low salinity (B) clearly resulted in more genes to be changed on a higher level compared to hypercapnia (A), where levels of change were mainly between a log_2_-ratio of -0.125 - 0.125. (C) Numbers of genes found to be regulated in expression simultaneously in response to a salinity dilution, as well as short-term hypercapnia (only transcripts with a log_2_-ratio > |0.200| were considered).

When compared to gene expression changes observed after 7 days of acclimation to a 2-fold reduction in salinity (from S = 32 to S = 15; [37]), short- and long-term acidification (10-fold increase in *p*CO_2_) resulted in smaller changes in expression levels in fewer transcripts: only 8 transcripts were regulated > 1.5-fold (log_2_-ratio > |0.60|) in the present study, while 533 transcripts were regulated > 1.5-fold in the low salinity acclimation study; maximum expression changes of up to 14-fold (log_2_-ratio = 3.8) were observed in the low salinity acclimation study (Figure 3B, 4). Nevertheless, a considerable amount of transcripts were regulated during acclimation to both abiotic stressors (table 2).

##### Comparison of qRT-PCR and microarray analysis

**Figure 5:**
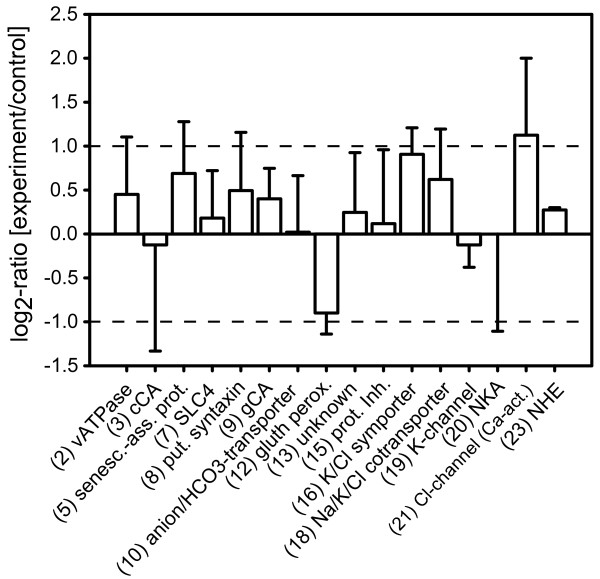
**Change of expression levels of distinct transcripts in gill 7 as response of *Carcinus maenas *to long-term exposure (11 weeks) to hypercapnia analysed by quantitative real-time polymerase chain reaction**. Bars represent the median log_2_-ratio and median deviation (error bars) of the respective gene. Numbers and abbreviations refer to primers and genes as used in the qRT-PCR (see Additional File 1 Table S2 for primer sequences and full names). No statistical significance was detected (sign-test with p < 0.05), but several transcripts exhibit a tendency to be up- or down-regulated, e.g. chloride associated transporters (no. 16, 18, 21).

In the long-term experiment, no significant changes in expression were found, likely due to the high biological variance within samples (coefficients of variation of the ratio 400 Pa/39 Pa CO_2 _between 15 to 79%, Figure 5). In the short-term experiment, 7 out of the 8 tested genes responded in the same direction as in the microarray analysis (Additional File 2 Figure S1). In one case (glycosyl-phosphatidylinositol-linked carbonic anhydrase VII [GenBank:DN739347.1]), the transcript was slightly, but significantly down-regulated in the microarray analysis, whereas it was up-regulated in the qRT-PCR analysis. Variation in both, qRT-PCR and in the microarray experiment was high.

##### Most strongly affected transcripts

The most down-regulated gene coded for a senescence-associated protein [GenBank:DV944570.1] in gill 7 on day 7 with a median log_2_-ratio of -1.16 ± 0.64 (2.2-fold change, sign test and F-test with p < 0.05, table 3). Its expression was not altered in the low-salinity experiment. An unknown transcript [GenBank:DN161290.1], suggested to encode for Syntaxin binding protein 2 by Towle et al. [37]), was the most strongly up-regulated transcript in gill 7 on day 7 with a median log_2_-ratio of +0.88 ± 0.36 (1.9-fold change, sign test and F-test with p < 0.05). This transcript was also the most strongly up-regulated gene in gill 8 in response to low salinity transfer [37]. Pg1 protein [GenBank:DW250369.1], only identified by F-test, was another highly up-regulated transcript on day 3, while it showed strong down-regulation on day 7. In gill 9, the most strongly down-regulated gene was identified on day 7 as a 23S ribosomal RNA gene [GenBank:DN796131.1] with a median log_2_-ratio of -0.87 ± 0.46 (1.9-fold change, sign test and F-test with p < 0.05). This gene was strongly up-regulated during the low-salinity experiment. Additionally, a different 23S ribosomal gene was found to be equally strongly affected on day 7 in gill 7 ([GenBank:DN635040.1], log_2_-ratio = 0.80 ± 0.22, 1.7-fold change, sign test with p < 0.05, not included in F-test). The most strongly up-regulated transcript in gill 9 at day 3 was a calcium-activated chloride channel [GenBank:DW584691.1] with a median log_2_-ratio of +0.81 ± 0.34 (1.7-fold change, sign test and F-test with p < 0.05). It was also the most highly up-regulated transcript in the long-term exposed animals (log_2_-ratio = 1.12 ± 0.88, 2.2-fold change), although not tested significant due to high variance between replicates. Additionally, rRNA intron-encoded homing endonuclease [GenBank:DV944193.1] was identified by F-test as being differentially expressed. Besides 4 unknown, but strongly changed transcripts (rank 8-11, table 2), a thymosin isoform was identified to be strongly down-regulated in gill 9 on day 3 (log_2_-ratio = -0.46 ± 0.14, 1.4-fold change, sign test with p < 0.05). In addition, a hyperpolarization-activated cyclic nucleotide-gated potassium channel (HCN2, [GenBank: DW250260.1]), was significantly down-regulated in both gills on day 7 (gill 7 with a log_2_-ratio = -0.46 ± 0.24/1.4-fold, and gill 9 with -0.14 ± 0.08/1.1-fold, sign test and F-test with p < 0.05) with a tendency for down-regulation in both gills on day 3. In the low-salinity study, it was only slightly down-regulated [37].

##### Acid/base and ion regulation

None of the acid-base regulatory candidate genes were regulated more than 1.3-fold in our microarray experiment at days 3 and 7. Of 9 candidate genes tested on the microarray, only 2 were significantly regulated with respect to controls. Among them were a Cl^-^/HCO_3_^- ^exchanger ([GenBank:CX994129.1], -1.1-fold, sign test and F-test with p < 0.05) and the glycosyl-phosphatidylinositol-linked carbonic anhydrase VII ([GenBank:DN739347.1], -1.1-fold, sign test and F-test with p < 0.05). In contrast, during acclimation to reduced salinity, Towle et al [37] found 5 of this 9 acid-base regulatory candidate genes to be altered by more than 1.3-fold, including the cytoplasmic carbonic anhydrase ([GenBank:DW251095.1], + 3.4-fold), the Na^+^/K^+^-ATPase alpha subunit ([GenBank:DW250552.1], + 2.6-fold), a member of the anion/bicarbonate transporter family (abts-3) ([GenBank:DN202373.1], similarity to a Na^+^/HCO_3_^- ^transporter (SLC4A11), - 2.4-fold), the glycosyl-phosphatidylinositol-linked carbonic anhydrase VII ([GenBank:DN739347.1], - 1.9-fold) and the Cl^-^/HCO_3_^- ^exchanger (SLC4A1; [GenBank:CX994129.1], + 1.3-fold).

In the long- term acclimation study (11 weeks), no significant changes in expression could be found, but variances were extremely high (coefficients of variation of the ratio 400 Pa/39 Pa CO_2 _ranged from 15 to 79%). Only in the case of the sodium-hydrogen exchanger NHE3, variance was smaller (1.3%).

## Discussion

Green crabs, especially those of the Baltic Sea, are known to be effective ion and acid-base regulators [25,49,50]. When exposed to hypercapnia, *Carcinus maenas *is characterized by a rapid pH_e _compensatory response that involves the accumulation of bicarbonate in its hemolymph ([17]). This high HCO_3_^- ^level is sustained even over a period of 11 weeks (Appelhans et al., under review). The enrichment of HCO_3_^- ^in the crabs' hemolymph may be mediated by altered ion regulation processes in the gills and thus can be expected to also leave a footprint in the gills' transcriptome. However, long-term hypercapnia incubations may additionally result in other physiological responses, pointing to trade-offs in energy allocation in favor of extra-/intracellular ion homeostasis vs. protein metabolism and growth, as has been concluded for molluscs, teleost fish and echinoderms [13,23,51-53]. We hypothesized that an increased demand for ion transport and modifications in epithelial CO_2 _permeability would influence expression patterns of the respective gene transcripts in gills in both, short- and long-term hypercapnia scenarios.

Variance-based analysis of the microarray identified a significant effect of *p*CO_2 _(53 vs. 440 Pa) on 26% of the transcripts. However, expression profiles in this study revealed that short-term hypercapnia (10-fold increase in seawater *p*CO_2_) does not act as a strong stressor on gill tissues. Expression changes were moderate and it has to be considered that some of the observed changes are random noise, given also that only few significant effects persisted over both time points (3 and 7 days) and considering the partially differing results from both statistical tests applied (40% of trancripts identified to be differentially expressed by both, sign test and variance-based analysis). Only relatively few transcripts (2% of all tested transcripts) were differentially regulated more than 1.3-fold, with maximum observed changes of 2.2-fold. Even these changes are much lower than those elicited by a 2-fold decrease in salinity (transfer from S = 32 to S = 15), where maximum observed changes in expression were 14-fold [37]. A negative effect of hypercapnia on transcripts related to energy metabolism, as has been shown in sea urchin larvae [53], was not observed in our study. Transcripts known to be involved in acid-base responses were not regulated strongly in response to elevated *p*CO_2_. Instead, new acid-base regulatory candidate genes were identified to be differentially expressed, including a calcium activated chloride channel, a multispanning membrane protein (transmembrane 9 superfamily protein member 4), a hyperpolarization activated cyclic nucleotide-gated potassium channel-2, tetraspanin 3 (transmembrane super family 4 member 8) and a potential integrin alpha-7.

### Short-term hypercapnia does not lead to a pronounced stress response

Only few stress-associated genes were differentially regulated in in gills in response to short-term hypercapnia. Therefore, short-term hypercapnia does not evoke a pronounced cellular stress-response (CSR) in the gills of adult *Carcinus maenas *from the Baltic Sea. A lack of pronounced changes in the expression of heat-shock proteins supports this interpretation, as does the finding that in general only small expression level changes occured. Heat-shock proteins (HSPs) are highly conserved among species and involved in acute stress responses towards various stressors [54]. Usually, cellular responses through HSP occur rapidly and transiently [55,56]. Thus, it cannot be excluded that a CSR occurred and ended before our first sampling after 3 days. However, the observation that a hypoosmotic acclimation with its much stronger changes in gene expression patterns also did not elicit a pronounced CSR (even within the first hours of exposure; [37]) corroborates the interpretation that the applied changes in seawater *p*CO_2 _do not trigger a prolonged or pronounced CSR in gills of *C. maenas*. As focus has been laid upon the gills, it should be considered that hypercapnia may indeed provoke a CSR in other organs.

Although magnitudes of change in gene expression were comparatively low (e.g. compared to [37,53]), we found indications for a re-arrangement of the green crabs' gill epithelia and support for specialization of each single posterior gill.

### Re-arrangement of the gill epithelium and epithelial cell membranes

Enrichment analysis supports that a reorganization of gill epithelia occurs during the initial stages (< 7 days) of hypercapnia acclimation. The GO-term "structural modification" is the only biological process that was enriched within up-regulated genes in response to hypercapnia. During salinity acclimation, posterior gills of *C. maenas *also undergo structural modification through extension of the apical plasma membrane infolding system of the thick prismatic salt-transporting epithelium and through an increase of the subcuticular space [50]. Although no genes encoding for cellular junction proteins such as claudins, occludins, cadherins or selectins were tested on the microarray used in the present study, our analysis identified several other genes potentially involved in structural rearrangements. A member of the tetraspanin family was found to be strongly down-regulated during short-term hypercapnia and slightly during low salinity acclimation [37]. Tetraspanins are a group of four-transmembrane-domain proteins that are expressed in epithelia and known to be involved in diverse cellular processes (e.g. morphogenetic re-organization of monolayers of epithelial cells [57]). They are generally described as molecular 'facilitators' or 'organizers' at the plasma membrane [58,59]. Furthermore, integrin-tetraspanin complexes play an important role in cell-cell-adhesion at cellular junctions [56,59,60]. A putative Integrin-alpha-7 was identified to be significantly and strongly down-regulated in the short-term acidification experiment. Due to their essential role in cell adhesion and cell-cell communication, biochemical functions of integrins are likely to be highly conserved in metazoans [61]. The common and distinct regulation of a tetraspanin and an integrin indicate that the possible complex of both could play a role during re-arrangement of the gill. Another strongly up-regulated transcript encodes for a multispanning endomembrane protein of the transmembrane 9 superfamily (protein member 4 = TM9SF4), also known as p76 in humans. TM9SF4 plays an important role in cellular adhesion, membrane reconfiguration and vesicle mediated transport [62,63]. It thus can be hypothesized that exposure to elevated seawater *p*CO_2 _has an influence on the membrane composition of gill epithelial cells, and on the cell composition of the epithelium of the gills of *C. maenas*. This warrants detailed studies on structural changes of the gills in response to hypercapnia (eg. light or electron microscopy and (immune-) histochemical investigations), including studies on the involvement of the three membrane proteins mentioned above.

### Specialization of the gills

As described above, only the three posterior gills 7 to 9 were found to be involved in ion- and acid-base regulation in *C. maenas*. The anterior six gill pairs are primarily important for gas exchange [17,26,27]. However, a more individualized specialization of each of the posterior gills has been shown for other species with respect to salinity changes [64,65]. For *C. maenas*, Siebers et al. [26] detected a salinity-dependent activation of Na^+^/K^+^-ATPase with increasing activity at decreasing salinities mainly in the posterior gills, but only subtle differences between the individual gills 7-9. Henry et al. [66] made a corresponding observation with respect to carbonic anhydrase. However, based on the distribution of V-type H^+^-ATPase in the posterior gills of several (intertidal) crab species, Tsai and Lin [65] showed that the functional differentiation in crab gills generally is not only between anterior and posterior, but also within individual gill lamellae. While the variance-based analysis of the data set suggested no significant effect of the factor "gill" on gene expression levels, enrichment analysis indicated different and specific responses to hypercapnia acclimation between posterior gills 7 and 9. Gill 7 responded stronger to hypercapnia than gill 9 with respect to both, the number of genes affected and the overall magnitude of change in gene expression. Most transcripts were either significantly regulated for one or the other gill, only 11% were regulated in parallel. The result of the enrichment analysis further suggested that structural changes are mainly associated with gill 9. However, the conflicting results of the two statistical methods used demand more detailed investigations on the cell ultrastructural level to substantiate potential differences in function between gills.

### Comparison with gene expression levels in response towards low salinity

When responses to hypercapnia (this study) and hypoosmotic acclimation [37] are compared, it becomes obvious that hyposmotic acclimation to a 2-fold reduction in salinity results in far larger expression changes than acclimation to a 10-fold increase in seawater *p*CO_2_. Nevertheless, acute hyposmotic acclimation also does not lead to a pronounced CSR [37]. *C. maenas *from the western Baltic Sea show an increased ability for hyperosmoregulation when exposed to low salinities (salinity < 20) than animals from the more saline North Sea (salinity > 30, [47]). Consequently, they also must possess a very high ion regulatory capacity and as salinity was low in our experiment (S = 15), the gill ion regulatory machinery was working at a comparatively high load. Microarray analysis only allows detection of relative changes in gene expression. If a certain transcript is already highly expressed, even a small relative change on the level of transcript expression could result in a highly effective regulatory capacity on the protein level. Even small changes may allow for successful acclimation. Furthermore, the strong fluctuations (both in rate and magnitude) in salinity, *p*CO_2 _and temperature observed in Kiel Fjord [19], might have led to an adaptation towards excess ion regulatory capacity that can be recruited upon demand. This recruitment might take place on the post-transcriptional level [23,67] and would therefore remain undetected in our microarray analysis.

### Old and new candidate genes for hypercapnia acclimation in crustaceans

In addition to the above discussed potential role of the multispanning membrane protein TM9SF4 in structural re-arrangement of gill epithelia, its hypercapnia induced up-regulation might be relevant for cellular acid-base regulation. TM9SF4 is known to participate in vesicular transport [68] and Schimmöller et al. [69] suggested its association with endosomes (acidic compartments/vesicles in mammalian cells). In posterior *C. maenas *gills, intracellular vesicles were postulated to be involved in cellular acid-base regulation via V-H^+^-ATPases [70].

A calcium-activated chloride channel (CaCC) was strongly up-regulated in gill 9 on day 3 and it was also up-regulated after 11 weeks of hypercapnia. Beside others, CaCCs have been shown to play a key role in epithelial secretion [71,72], but a high variability within this class of channels with respect to physiological roles and mechanisms of regulation was observed [73,74]. A member of a CaCC subfamily has also been shown to act in cell-cell adhesion through interaction with an integrin (e.g. in lung cancer [67], reviewed [75]). Chloride channels have been hypothesized to be situated in the basolateral membrane of epithelial cells in gills of osmoregulating crabs [32,33]. The identified CaCC as described above might be an important candidate gene in Cl^- ^regulation. In order to achieve electroneutrality, Cl^- ^typically is the counter-ion of HCO_3_^- ^during the extracellular pH regulatory reaction. Extracellular HCO_3_^- ^accumulation is probably enabled by Cl^-^/HCO_3_^- ^exchangers [20]. While respective Cl^-^/HCO_3_^- ^exchangers (AE) have been postulated to be situated in the apical membrane in crustaceans, molecular identification and/or biochemical characterization is still lacking. On the other hand, a basolateral-situated AE can be discussed to play a role in pH and volume regulation [33]. In acid secretion, a respective exchanger is postulated to transport HCO_3_^- ^ions from the cell into the hemolymph in exchange for Cl^- ^ions and therefore is argued to sit in the basolateral membrane. As has been postulated in fish, other transporters in close proximity to a Cl^-^/HCO_3_^- ^exchanger can be discussed to favor the electroneutral exchange of Cl^- ^and HCO_3_^- ^against an unfavorable Cl^- ^gradient [76]. A Cl^-^-channel like the identified CaCC could be an important additional player and facilitator in this Cl^-^/HCO_3_^- ^exchange.

Additionally, a hyperpolarization-activated cyclic nucleotide-gated potassium channel (HCN) was significantly down-regulated in the short-term hypercapnia study. So far, HCNs (1+4) have been shown to be situated in the plasma membrane of vallate papillea taste cells in rat tongue. Those transporters/receptors are associated with the basolateral membrane and play a role in response to sour stimuli (extracellular protons) by mediating an inwardly directed current [77]. On the other hand, lowered intracellular pH has been shown to lead to a decreased opening speed of the channel in thalamocortical neurons of the rat ventrobasal thalamic complex [78]. Thus, an altered extracellular acid-base status might interact with the function of this protein.

Future studies should characterize these candidate genes with respect to localization and function. In the case of the CaCC and HCN, electrophysiological experiments could reveal in which way these transporters mediate ion fluxes across the gill epithelium.

Several transporters and channels shown to be involved in ion or acid-base regulation during hypercapnia acclimation in other studies on diverse marine organisms [33,51,79] were not affected in *C. maenas *gill tissue. The sodium pump, Na^+^/K^+^-ATPase (NKA), is crucial for the maintenance of ion gradients that drive acid-base regulation [80]. This primary active transporter is a key player in establishing the characteristic ion gradient that is used by many secondary active transporters. NKA transcript and protein level, as well as activity increased in response to decreased salinities in diverse crabs, including *C. maenas *[*C. maenas*: 37, 69; others: 64, 65, 81]. During hypercapnia acclimation, Deigweiher et al. [52] documented that in teleost fish, NKA mRNA concentration decreased initially (day 4), only to increase 2-fold after 6 weeks of exposure to a *p*CO_2 _of 1 kPa. In contrast, O'Donnell et al. [53] found that in sea urchin larvae, NKA mRNA was down-regulated in response to hypercapnia. Although *NKA *expression decreased significantly in cephalopod embryos and hatchlings exposed to a *p*CO_2 _of 0.4 kPa, no change in expression was detected in juveniles under comparable conditions [23]. We did not find changes in *NKA *expression in *C. maenas *gill tissue in the present study. Only one Cl^-^/HCO_3_^-^-anion exchanger from the SLC family 4 (member 1; [GenBank:CX994129.1]) was significantly down-regulated in the short-term experiment (1.1-fold), while a different anion-bicarbonate exchanger, similar to the SLC family 4, member 11 [GenBank:DN202373.1]), and a vacuolar H^+^-ATPase [GenBank:DY656042.1] were not affected. Another important transporter for acid-base regulation, a Na^+^/H^+ ^exchanger (NHE3) was not significantly regulated in *C. maenas *gill tissue in the long- term experiment. *C. maenas *also possesses two branchial isoforms of carbonic anhydrase (CA) in its posterior gills, a membrane-associated and a cytoplasmic form [82]. The enzyme catalyzes the highly energy-demanding transition of H_2_O and CO_2 _to HCO_3_^- ^and H^+ ^and vice versa. It is of great importance in osmoregulation (especially the cytoplasmic pool [66]), acid-base regulation and CO_2 _excretion in the gills of crustaceans [71,83]. However in this study, no response of CAs was observed in short-term hypercapnia experiments, except for a slight significant down-regulation of glycosyl-phosphatidylinositol-linked carbonic anhydrase VII [GenBank:DN739347.1] in both, gill 7 and 9, on day 7. In agreement with the results of the short-term experiment, we also found no significant expression changes following long-term exposure to hypercapnia.

We suggest that the ion regulatory apparatus of Baltic *C. maenas *already works at a high load due to the demands of a hyposmotic habitat with large fluctuations in *p*CO_2_. Therefore, only moderate mRNA expression level changes might be necessary to compensate hypercapnia in *C. maenas*. Compensation of the ion regulatory apparatus might additionally take place on the post-transcriptional level or is facilitated by transcripts not included on this microarray. It has to be considered though, that to some extend, effects might be hidden under the experimental error. Nevertheless, those few genes for which we have identified relatively strong changes in expression levels are particularly interesting. They are likely key players of hypercapnia acclimation of crustacean gill tissues.

## Conclusions

The response of *Carcinus maenas *to elevated seawater *p*CO_2 _based on the gill transcriptome does not suggest that seawater acidification acts as a strong stressor for the western Baltic population of this species. Following short-term hypercapnia, the low but specific responses of the gills indicate that (1) the response to hypercapnia is partially similar to the response to hypoosmotic conditions; (2) a multispanning membrane protein (TM9SF4), a calcium-activated chloride channel (CaCC) and a hyperpolarization-activated cyclic nucleotide-gated potassium channel (HCN) are strongly regulated in response to hypercapnia and may be involved in acid-base regulation; (3) posterior gills might respond differently to hypercapnia; and (4) a re-configuration of the epithelial gill membrane, including the involvement of a tetraspanin-integrin complex, might occur.

Due to the low salinity environment in the Baltic Sea in this hypercapnia study, effects on distinct gene expression levels to one of the respective abiotic factors might be influenced by the other. It is therefore essential to study both abiotic factors separately and in more detail. Here, potential pathological effects of the long-term consequences of elevated blood bicarbonate concentrations on various tissues and organs should also deserve particular attention. We also expect that stronger effects of hypercapnia are to be found on the proteome and metabolome level.

## Authors' contributions

SF carried out the short-term incubations, microarray experiments and real-time PCR, analyzed the results and drafted the manuscript. Most of the facts displayed here are content of SF's Diploma thesis at the IFM-GEOMAR, Kiel, Germany 2010. RK contributed to the analysis, illustration and interpretation of the data and the preparation of the manuscript. YA carried out the long term incubation experiments and participated in the sampling procedure. DWT passed away during the preparation of the manuscript; before his untimely death, he significantly contributed to the microarray and study design and coordination, and provided the microarray facilities. FM and MZ conceived the study, participated in its design, coordination and analysis, supplied infrastructure and material and helped to draft the manuscript. All authors read and approved the final manuscript.

## Supplementary Material

Additional File 1 Tables S1-S6.
**Table S1. Annotation details on transcripts for the *Carcinus maenas *the microarray assay applied in this study**. Annotation results on transcripts for the Carcinus maenas microarray assay applied in this study from a newly performed bioinformatic analysis conducted with the internet portal Blast2GO [43] in 2009 by the authors of this study, compared to a first annotation provided by Towle et al. [37]. Included are the sequence length of the aligned sequence (= seq. length), the number of hits for the transcript (= #hits), the mean similarity of the alignment (= mean sim), the number of Gene Ontology (GO) terms assigned to that blast hit (= #GOs), a description of the assigned GOs (= GOs), Enzyme Codes (= EC), results of the InterProScan, the exact hit description of the best hit, as well as the accession number (= hit ACC, E-value, similarity, score, alignment length and number of positive matches bases of the best hit.
**Table S2. Details on primers for quantitative real-time polymerase chain reaction (qRT-PCR) to assess responses of *Carcinus maenas *to hypercapnia**. Primer sequences (5' → 3') and descriptions of the targeted genes used in the real-time polymerase chain reaction (qRT-PCR) in the short- and long-term hypercapnia experiments on *Carcinus maenas*. Numbers ('no.') are according to the numbers used in Figure 4 and Additional File 2 Figure S1. Accession numbers (ACC. no.) refer to the ESTs generated for the *C. maenas *microarray by Towle et al. [37] and the database GenBank (NCBI). R^2 ^and efficiency were tested in a qRT-PCR dilution series (for details, see Material & Methods).
**Table S3. Transcripts significantly regulated in response to hypercapnia in *Carcinus maenas***. Transcripts significantly regulated in response to hypercapnia in *Carcinus maenas *as identified by variance-based linear modelling (F-test) and sign test. Out of 1634 genes, 678 were identified by sign test, 578 genes were identified by F-test, and 378 were identified to be affected by both statistical tests. Transcripts are sorted alphabetically after the accession number (ACC. no., database GenBank (NCBI)). Bold and underlined = significantly regulated as identified by sign test, p < 0.05; bold = significantly regulated as identified by both statistical tests (sign test and F test, p < 0.05); italic = differentially expressed as identified by F-Test. Accession numbers refer to the ESTs generated for the *C. maenas *microarray by Towle et al. [37]. Values are given as median and median deviation (MD).
**Table S4. Significantly regulated transcripts associated with a cellular stress-response in *Carcinus maenas *when exposed to hypercapnia**. Significantly regulated transcripts in the short-term hypercapnia study on *Carcinus maenas *associated with a cellular stress-response acoording to [38]. Bold and underlined (values) = significantly regulated (sign test, p < 0.05), bold (ACC. no.) = transcripts identified to be significantly regulated by both tests (sign test and F-test, p < 0.05). Accession numbers (ACC. no.) refer to the GenBank database (NCBI).
**Table S5. Significantly up-regulated transcripts in the short-term hypercapnia study associated with structural molecule activity in gills of *Carcinus maenas***. Transcripts identified to be responsible for the over-representation of the GO-term "structural modification" (GO:0005198) in *Carcinus maenas *gills exposed to hypercapnia (enrichment analysis with Fisher's exact test). Bold and underlined (values) = significantly regulated as identified by sign test (p < 0.05); bold (ACC. no.) = identified to be significantly regulatd by both tests (sign test and F-test, p < 0.05). Accession numbers (ACCs) refer to the database GenBank (NCBI).
**Table S6. Details on transcripts of special interest identified by microarray analysis on gills of *Carcinus maenas *after exposure to short-term hypercapnia**. Identification and details on transcripts of special interest identified by microarray analysis, after an additional NCBI blastx. * = NCBI blastn; ** = see Towle et al. [37] for details. Accession numbers (ACC. no.) refer to the database GenBank (NCBI). Click here for file

Additional file 2 **figure S1. Comparison of distinct transcripts of the microarray analysis vs. qRT-PCR for the response of *Carcinus maenas *to short-term hypercapnia**. Comparison of the regulation of distinct transcripts of gill 9 for the *Carcinus maenas *response to short-term hypercapnia (1 week, April 2009) in the microarray analysis with results of the qRT-PCR experiment performed on the respective genes from the short-term incubation conducted in April 2010. In 7 of 8 cases, both techniques show the same tendency in regulation. Values represent median log_2_-ratios with median deviation (error bars). Transcript numbers according to Additional File 1 Table S2. Senesc. ass. prot = senescence-associated protein, put. syntaxin = putative Syntaxin binding protein 2, gCA = glycosyl-phosphatidylinositol-linked carbonic anhydrase VII, prot. inh. = hemozyte kazal-type proteinase inhibitor, K-channel = hyperpolarization activated cyclic nucleotide-gated potassium channel 2, NKA = Na^+^/K^+^-ATPase alpha subunit, Cl-channel (Ca-act.) = calcium acitvated chloride channel. Click here for file

## References

[B1] Caldeira K Ocean model predictions of chemistry changes from carbon dioxide emissions to the atmosphere and ocean Journal of Geophysical Research 2005 110 C9 1 12

[B2] IPCC Climate Change 2007: The Physical Science Basis Changes 2007 1 21

[B3] Tripati AK Roberts CD Eagle RA Coupling of CO2 and Ice Cheet Stability Over Major Climate Transitions of the Last 20 Million Years Science 2009 326 1394 1397 10.1126/science.1178296 19815724

[B4] Fabry V Seibel B Feely R Orr J Impacts of ocean acidification on marine fauna and ecosystem processes ICES Journal of Marine Science 2008 65 414 432 10.1093/icesjms/fsn048

[B5] Melzner F Gutowska M Langenbuch M Physiological basis for high CO2 tolerance in marine ectothermic animals: pre-adaptation through lifestyle and ontogeny? Biogeosciences 2009 6 4993 4738 10.5194/bgd-6-4993-2009

[B6] Doney SC Fabry VJ Feely RA Kleypas JA Ocean acidification: the other CO_2 _problem Annual Review of Marine Science 2009 169 192 10.1146/annurev.marine.010908.163834 21141034

[B7] Kroecker KJ Kordas RL Crim RN Singh GG Meta-analysis reveals negative yet variable effects of ocean acidification on marine organisms Ecology Letters 2010 13 1419 1434 10.1111/j.1461-0248.2010.01518.x 20958904

[B8] Michaelidis B Ouzounis C Paleras A Pörtner H Effects of long-term moderate hypercapnia on acid-base balance and growth rate in marine mussels Mytilus galloprovincialis Marine Ecology Progress Series 2005 293 109 118

[B9] Kurihara H Effects of CO2-driven ocean acidification on the early developmental stages of invertebrates Marine Ecology Progress Series 2008 373 275 284

[B10] Munday PL Jones GP Pratchett MS Williams AJ Climate change and the future for coral reef fishes Fish and Fisheries 2008 9 261 285 10.1111/j.1467-2979.2008.00281.x

[B11] Munday PL Dixsona DL Donelsona JM Jonesa GP Pratchett MS Devitsina GV Døving KB Ocean acidification impairs olfactory discrimination and homing ability of a marine fish PNAS 2009 106 6 1848 1852 10.1073/pnas.0809996106 19188596 PMC2644126

[B12] Ries JB Cohen AL McCorkle DC Marine calcifiers exhibit mixed responses to CO2-induced ocean acidification Geology 2009 37 12 1131 1134 10.1130/G30210A.1

[B13] Thomsen J Melzner F Moderate seawater acidification does not elicit long-term metabolic depression in the blue mussel Mytilus edulis Mar Biol 2010 157 2667 2676 10.1007/s00227-010-1527-0

[B14] Dupont S Ortega-Martinez O Thorndyke MC Impact of near-future ocean acidification on echinoderms Ecotoxicology 2010 19 449 462 10.1007/s10646-010-0463-6 20130988

[B15] Gutowska M Melzner F Pörtner H Meier S Cuttlebone calcification increases exposure to elevated seawater pCO2 in the cephalopod Sepia officinales Marine Biology 2010 157 7 1653 1663 10.1007/s00227-010-1438-0

[B16] Checkley DM Jr Dickson AG Takahashi M Radich A Eisenkolb N Asch R Elevated CO2 Enhances Otolith Growth in Young Fish Science 2009 324 1683 10.1126/science.1169806 19556502

[B17] Truchot J Blood acid-base changes during experimental emersion and reimmersion of the intertidal crab Carcinus maenas (L.) Respiration Physiology 1975 23 351 360 10.1016/0034-5687(75)90086-9 238271

[B18] Dejours P Beekenkamp H Crayfish respiration as a function of water oxygenation Respiration Physiology 1977 30 241 251 10.1016/0034-5687(77)90033-0 17899

[B19] Thomsen J Gutowska MA Saphörster J Heinemann A Trübenbach K Fietzke J Hiebenthal C Eisenhauer A Körtzinger A Wahl M Melzner F Calcifying invertebrates succeed in a naturally CO2 enriched coastal habitat but are threatened by high levels of future acidification Biogeosciences 2010 7 5119 5156 10.5194/bgd-7-5119-2010

[B20] Larsen B Pörtner H Jensen F Extra- and intracellular acid-base balance and ionic regulation in cod (Gadus morhua) during combined and isolated exposures to hypercapnia and copper Marine Biology 1997 128 2 337 346 10.1007/s002270050099

[B21] Spicer JI Raffo A Widdicombe S Influence of CO2-related seawater acidification on extracellular acid-base balance in the velvet swimming crab Necora puber Marine Biology 2007 151 3 1117 1125 10.1007/s00227-006-0551-6

[B22] Gutowska M Melzner F Langenbuch M Acid-base regulatory ability of the cephalopod (Sepia officinalis) in response to environmental hypercapnia Journal of Comparative Physiology, Part B 2009 180 3 323 335 10.1007/s00360-009-0412-y 19838713

[B23] Hu MY Tseng Y-C Stumpp M Gutowska MA Kiko R Lucassen M Melzner F Elevated seawater *p*CO2 differentially affects branchial acid-base transporters over the course of development in the cephalopod *Sepia officinalis* Am J Physiol Regul Integr Comp Physiol 2011 300 5 R1100 R1114 10.1152/ajpregu.00653.2010 21307359

[B24] Gilmour KM Perry SF Carbonic anhydrase and acid-base regulation in fish The Journal of Experimental Biology 2009 212 1647 1661 10.1242/jeb.029181 19448075

[B25] Truchot J Mechanisms of the compensation of blood respiratory acid-base disturbances in the shore crab, Carcinus maenas (L.) The Journal of Experimental Zoology 1979 210 3 407 416 10.1002/jez.1402100305

[B26] Siebers D Leweck K Markus H Winkler A Sodium Regulation in the Shore Crab Carcinus maenas as Related to Ambient Salinity Marine Biology 1982 69 37 43 10.1007/BF00396958

[B27] Cieluch U Anger K Aujoulat F Buchholz F Charmantier-Daures M Charmantier G Ontogeny of osmoregulatory structures and functions in the green crab Carcinus maenas (Crustacea, Decapoda) The Journal of Experimental Biology 2004 207 2 325 336 10.1242/jeb.00759 14668316

[B28] Storey KB Suspended animation: the molecular basis of metabolic depression Canadian Journal of Zoology 1988 66 1 124 132 10.1139/z88-016

[B29] Dalla Via J Van den Thillart G Cattani O De Zwaan A Influence of long-term hypoxia exposure on the energy metabolism of Solea solea. II. Intermediary metabolism in blood, liver and muscle Mar ecol Prog Ser 1994 111 1-2 17 27

[B30] Hochachka PW Oxygen -A key regulatory metabolite in metabolic defense against hypoxia Amer Zool 1997 37 595 603

[B31] Wu RSS Hypoxia: from molecular responses to ecosystem responses Marine Pollution Bulletin 2002 45 1-12 35 45 10.1016/S0025-326X(02)00061-9 12398365

[B32] Towle DW Weihrauch D Osmoregulation by Gills of Euryhaline Crabs: Molecular Analysis of Transporters American Zoology 2001 41 4 770 780 10.1668/0003-1569(2001)041[0770:OBGOEC]2.0.CO;2

[B33] Freire C Onken H McNamara J A structure-function analysis of ion transport in crustacean gills and excretory organs Comparative Biochemistry and Physiology, Part A 2008 151 3 272 304 10.1016/j.cbpa.2007.05.008 17604200

[B34] Storey KB Phosphofructokinase from foot muscle of the whelk, *Busycotypus canaliculatum*: Evidence for covalent modification of the enzyme during anaerobiosis Archives of Biochemistry and Biophysics 1984 235 2 665 672 10.1016/0003-9861(84)90242-X 6240229

[B35] Rahman MS Storey KB Role of covalent modification in the control of glycolytic enzymes in response to environmental anoxia in goldfish J Comp Physiol B 1988 157 813 820 10.1007/BF00691013

[B36] Kotlyar S Weihrauch D Paulsen RS Towle DW Expression of arginine kinase enzymatic activity and mRNA in gills of the euryhaline crabs *Carcinus maenas *and *Callinectes sapidus* The Journal of Experimental Biology 2000 203 2395 2404 31 10903154 10.1242/jeb.203.16.2395

[B37] Towle DW Henry RP Terwilliger NB Microarray-detected changes in gene expression in gills of green crabs (Carcinus maenas) upon dilution of environmental salinity Comparative Biochemistry and Physiology, Part D 2011 6 2 115 25 21220218 10.1016/j.cbd.2010.11.001

[B38] Kültz D Molecular and Evolutionary Basis of the Cellular Stress Response Annu Rev Physiol 2005 67 225 57 10.1146/annurev.physiol.67.040403.103635 15709958

[B39] Dickson AG Millero FJ A comparison of the equilibrium-constants for the dissociation of carbonic-acid in seawater media Deep-Sea Res 1987 34 1733 1743 10.1016/0198-0149(87)90021-5

[B40] Lewis E Wallace DWR Program Developed for CO_2 _System Calculations ORNL/CDIAC-105. Carbon Dioxide Information Analysis Center, Oak Ridge National Laboratory, U.S. Department of Energy, Oak Ridge, Tennessee 1998

[B41] Roy RN Roy LN Vogel KM Porter-Moore C Pearson T Good CE Millero FJ Campbell DM The dissociation constants of carbonic acid in seawater at salinities 5 to 45 and temperatures 0 to 45°C Marine Chemistry 1993 44 249 267 10.1016/0304-4203(93)90207-5

[B42] Dickson AG Standard potential of the reaction AgCl_s_+1*/*2H_2g _= Ag_s_+HCl_aq _and the standard acidity constant of the ion HSO_4_^- ^in synthetic sea-water from 273.15 K to 318.15 K J Chem Thermodyn 1990 22 113 127 10.1016/0021-9614(90)90074-Z

[B43] Blast2GO http://www.blast2go.org/

[B44] Conesa A Götz S Blast2GO Tutorial Interface 2009 Valencia, Spain

[B45] Jensen J Ørntoft T Normalization of real-time quantitative RT-PCR data: a model based variance estimation approach to identify genes suited for normalization - applied to bladder- and colon-cancer data-sets Cancer Research 2004 64 5245 5250 10.1158/0008-5472.CAN-04-0496 15289330

[B46] Andersen CL Jensen JL Ørntoft TF Normalization of Real-Time Quantitative Reverse Transcription-PCR Data: A Model-Based Variance Estimation Approach to Identify Genes Suited for Normalization, Applied to Bladder and Colon Cancer Data Sets Cancer Res 2004 64 5245 5250 10.1158/0008-5472.CAN-04-0496 15289330

[B47] Cui X Hwang JT Qiu J Blades NJ Churchill GA Improved statistical tests for differential gene expression by shrinking variance components estimates Biostatistics 2005 6 59 75 10.1093/biostatistics/kxh018 15618528

[B48] Storey JD A direct approach to false discovery rates J R Stat Soc B 2002 64 479 498 10.1111/1467-9868.00346

[B49] Theede H Ponat A Hiroki K Schlieper C Studies on the resistance of marine bottom invertebrates to oxygen-deficiency and hydrogen sulphide Marine Biol 1969 2 325 337 10.1007/BF00355712

[B50] Compere P Wanson S Pequeux A Gilles R Goffinet G Ultrastructural Changes in the Gill Epithelium of the Green Crab Carcinus maenas in Relation to the External Salinity Tissue & Cell 1989 21 2 299 318 18620265 10.1016/0040-8166(89)90073-6

[B51] Langenbuch M Poertner HO Energy budget of hepatocytes from Antarctic fish (*Pachycara brachycephalum and Lepidonotothen kempi*) as a function of ambient CO_2_: pH-dependent limitations of cellular protein biosynthesis? The Journal of experimental biology 2003 206 3895 3903 10.1242/jeb.00620 14555731

[B52] Deigweiher K Koschnick N Pörtner H Lucassen M Acclimation of ion regulatory capacities in gills of marine fish under environmental hypercapnia Am J Physiol Regulatory Integrative Comp Physiol 2008 295 R1660 R1670 10.1152/ajpregu.90403.2008 18799636

[B53] O'Donnell MJ Todgham AE Sewell MA Hammond LM Ruggiero K Fangue NA Zippay ML Hofmann GE Ocean acidification alters skeletogenesis and gene expression in larval sea urchins Mar Ecol Prog Ser 2010 398 157 171

[B54] Feder ME Hofmann GE Heat shock proteins, molecular chaperones, and the stress response: evolutionary and ecological physiology Annu Rev Physiol 1999 61 243 282 10.1146/annurev.physiol.61.1.243 10099689

[B55] Amrani M Corbett J Boateng SY Dunn MJ Yacoub MH Kinetics of Induction and Protective Effect of Heat-Shock Proteins After Cardioplegic Arrest Ann Thorac Surg 1996 61 1407 1411 10.1016/0003-4975(96)00085-9 8633950

[B56] Tomanek L Somero GN Time Course and Magnitude of Synthesis of Heat-Shock Proteins in Congeneric Marine Snails (Genus *Tegula*) from Different Tidal Height Physiological and Biochemical Zoology 2000 73 2 249 256 10.1086/316740 10801403

[B57] Yáñez-Mó M Tejedor R Rousselle P Sánchez -Madrid F Tetraspanins in intercellular adhesion of polarized epithelial cells: spatial and functional relationship to integrins and cadherins Journal of Cell Science 2001 114 Pt 3 577 87 11171326 10.1242/jcs.114.3.577

[B58] Berditchevski F Complexes of tetraspanins with integrins: more than meets the eye Journal of Cell Science 2001 114 Pt 23 4143 51 11739647 10.1242/jcs.114.23.4143

[B59] Zhang F Kotha1 J Jennings LK Zhang XA Tetraspanins and vascular functions Cardiovascular Research 2009 83 7 15 10.1093/cvr/cvp080 19251723 PMC2695701

[B60] Tiwari-Woodruff SK Buznikov AG Vu TQ OSP/claudin-11 forms a complex with a novel member of the tetraspanin super family and beta1 integrin and regulates proliferation and migration of oligodendrocytes The Journal of Cell Biology 2001 153 2 295 305 10.1083/jcb.153.2.295 11309411 PMC2169454

[B61] Takada Y Ye X Simon S The integrins Genome Biology 2007 8 215 10.1186/gb-2007-8-5-215 17543136 PMC1929136

[B62] Benghezal M Cornillon S Gebbie L Synergistic Control of Cellular Adhesion by Transmembrane 9 Proteins Molecular Biology of the Cell 2003 14 2890 2899 10.1091/mbc.E02-11-0724 12857872 PMC165684

[B63] Bergeret E Perrin J Williams M Grunwald D Engel E Thevenon D Taillebourg E Bruckert F Cosson P Fauvarque MO TM9SF4 is required for *Drosophila *cellular immunity via cell adhesion and phagocytosis Journal of Cell Science 2008 121 3325 3334 10.1242/jcs.030163 18796536

[B64] Jayasundara N Towle DW Weihrauch D Spanings-Pierrot C Gill-specific transcriptional regulation of Na+/K+-ATPase -subunit in the euryhaline shore crab *Pachygrapsus marmoratus*: sequence variants and promoter structure The Journal of Experimental Biology 2007 210 2070 2081 10.1242/jeb.004309 17562880

[B65] Tsai J Lin H V-type H+-ATPase and Na+, K+-ATPase in the gills of 13 euryhaline crabs during salinity acclimation The Journal of Experimental Biology 2007 210 Pt 4 620 627 17267648 10.1242/jeb.02684

[B66] Henry R Gehnrich S Weihrauch D Towle D Salinity-mediated carbonic anhydrase induction in the gills of the euryhaline green crab, Carcinus maenas Comparative Biochemistry and Physiology, Part A 2003 136 243 258 10.1016/S1095-6433(03)00113-2 14511744

[B67] Ramnanan CJ Storey KB Suppression of Na+/K+-ATPase activity during estivation in the land snail *Otala lactea* J Exp Biol 2006 209 677 688 10.1242/jeb.02052 16449562

[B68] Diaz E Schimmoller F Pfeffer SR A novel Rab9 effector required for endosome-to-TGN transport J Cell Biol 1997 138 283 290 10.1083/jcb.138.2.283 9230071 PMC2138197

[B69] Schimmöller F Díaz E Mühlbauer B Pfeffer SR Characterization of a 76 kDa endosomal, multispanning membrane protein that is highly conserved throughout evolution Gene 1998 216 2 311 318 10.1016/S0378-1119(98)00349-7 9729438

[B70] Weihrauch D Ziegler A Siebers D Towle D Active ammonia excretion across the gills of the green shore crab Carcinus maenas: participation of Na +/K + -ATPase, V-type H + -ATPase and functional microtubules The Journal of Experimental Biology 2002 205 2765 2775 12177142 10.1242/jeb.205.18.2765

[B71] Suzuki M The *Drosophila *tweety family: molecular candidates for large-conductance Ca2+-activated Cl- channels Exp Physiol 2006 91 141 147 16219661 10.1113/expphysiol.2005.031773

[B72] Hartzell CH Yu K Xiao Q Chien LT Qu Z Anoctamin/TMEM16 family members are Ca2+-activated Cl- channels J Physiol 2009 587 10 2127 2139 10.1113/jphysiol.2008.163709 19015192 PMC2697287

[B73] Hartzell C Putzier I Arreola J Calcium-activated chloride channels Annual Review of Physiology 2005 67 719 58 10.1146/annurev.physiol.67.032003.154341 15709976

[B74] Abdel-Ghany M Cheng HC Elble RC Pauli BU The Breast Cancer β4 Integrin and Endothelial Human CLCA2 Mediate Lung Metastasis The journal of biological chemistry 2001 276 6 25438 25446 11320086 10.1074/jbc.M100478200

[B75] Eggermont J Calcium-activated Chloride Channels: (Un)known, (Un)loved? Proc Am Thorac Soc 2004 22 27 10.1513/pats.2306010 16113407

[B76] Hwang PP Perry SF Ionic and acid-base regulation Fish Physiology 2010 29 311 344

[B77] Stevens DR Seifert R Bufe B Müller F Kremmer E Gauss R Meyerhoff W Kaupp UB Lindemann B Hyperpolarization-activated channels HCN1 and HCN4 mediate responses to sour stimuli Nature 2001 413 631 635 10.1038/35098087 11675786

[B78] Munsch T Pape HC Modulation of the hyperpolarization-activated cation current of rat thalamic relay neurones by intracellular pH Journal of Physiology 1999 519 2 493 504 10.1111/j.1469-7793.1999.0493m.x 10457065 PMC2269522

[B79] Todgham AE Hofmann GE Transcriptomic response of sea urchin larvae Strongylocentrotus purpuratus to CO2-driven seawater acidification J Exp Biol 2009 212 2579 2594 10.1242/jeb.032540 19648403

[B80] Ahearn GA Mandal PK Mandal A Calcium regulation in crustaceans during the molt cycle: a review and update Comparative Biochemistry and Physiology Part A 2004 137 247 257 10.1016/j.cbpb.2003.10.012 15123199

[B81] Lucu C Towle D Na+ + K+ -ATPase in gills of aquatic crustacea Comparative Biochemistry and Physiology, Part A 2003 135 195 214 10.1016/S1095-6433(03)00064-3 12781821

[B82] Serrano L Henry R Differential expression and induction of two carbonic anhydrase isoforms in the gills of the euryhaline green crab, Carcinus maenas, in response to low salinity Comparative Biochemistry and Physiology, Part D 2008 3 2 186 193 20483218 10.1016/j.cbd.2008.02.003

[B83] Burnett L Dunn T Infantino R Jr Gilles R, Gilles-Baillien M The Function of Carbonic Anhydrase in Crustacean Gills Transport Processes, Iono- and Osmoregulation Heidelberg: Springer-Verlag 1985 159 1

