# Supplementary figures and images for: Effects of elevated seawater pCO2 on gene expression patterns in the gills of the green crab, Carcinus maenas
Source: BMC Genomics. 2011 Oct 6;12:488. doi: 10.1186/1471-2164-12-488 (PMC3206878; doi:10.1186/1471-2164-12-488)

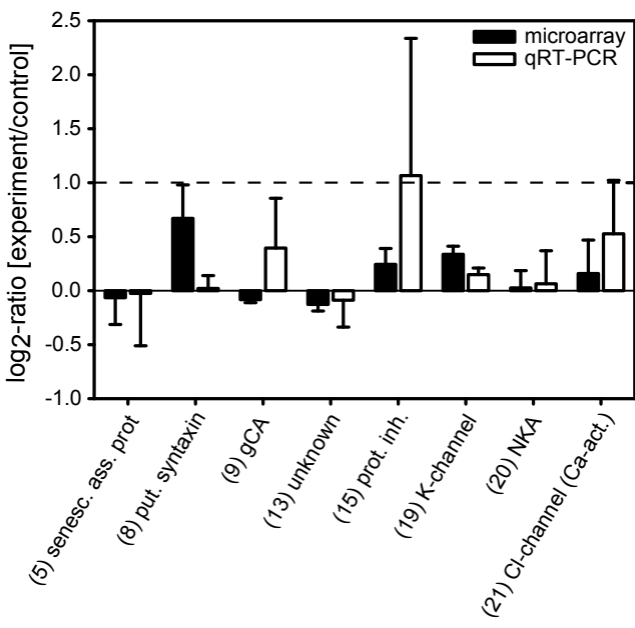

Supplement: Additional file 2 — figure S1. Comparison of distinct transcripts of the microarray analysis vs. qRT-PCR for the response of Carcinus maenas to short-term hypercapnia. Comparison of the regulation of distinct transcripts of gill 9 for the Carcinus maenas response to short-term hypercapnia (1 week, April 2009) in the microarray analysis with results of the qRT-PCR experiment performed on the respective genes from the short-term incubation conducted in April 2010. In 7 of 8 cases, both techniques show the same tendency in regulation. Values represent median log2-ratios with median deviation (error bars). Transcript numbers according to Additional File 1 Table S2. Senesc. ass. prot = senescence-associated protein, put. syntaxin = putative Syntaxin binding protein 2, gCA = glycosyl-phosphatidylinositol-linked carbonic anhydrase VII, prot. inh. = hemozyte kazal-type proteinase inhibitor, K-channel = hyperpolarization activated cyclic nucleotide-gated potassium channel 2, NKA = Na+/K+-ATPase alpha subunit, Cl-channel (Ca-act.) = calcium acitvated chloride channel. [file 1471-2164-12-488-S2.PDF]
